# Robotic complete mesocolic excision with central vascular ligation for right colonic tumours – a propensity score-matching study comparing with standard laparoscopy

**DOI:** 10.1093/bjsopen/zrab016

**Published:** 2021-04-08

**Authors:** J S Khan, A Ahmad, M Odermatt, D G Jayne, N Z Ahmad, N Kandala, N P West

**Affiliations:** 1 Department of Colorectal Surgery, Queen Alexandra Hospital, Portsmouth, UK; 2 School of Health, Education, Medicine and Social Care, Anglia Ruskin University, Chelmsford, UK; 3 Department of Surgery, Triemli City Hospital, Zurich, Switzerland; 4 Leeds Institute of Medical Research at St James's, University of Leeds, Leeds, UK; 5 Faculty of Sciences, School of Health & Care Professions, University of Portsmouth, UK; 6 Pathology & Data Analytics, Leeds Institute of Medical Research at St. James’s, University of Leeds, UK

## Abstract

**Background:**

Laparoscopic complete mesocolic excision (CME) of the right colon with central vascular ligation (CVL) is a technically demanding procedure. This study retrospectively evaluated the feasibility, safety and oncological outcomes of the procedure when performed using the da Vinci^®^ robotic system.

**Methods:**

A prospective case series was collected over 3 years for patients with right colonic cancers treated by standardized robotic CME with CVL using the superior mesenteric vessels first approach. The CME group was compared to a 2 : 1 propensity score-matched non-CME group who had conventional laparoscopic right colectomy with D2 nodal dissection. Primary outcomes were total lymph node harvest and length of specimen. Secondary outcomes were operative time, postoperative complications, and disease-free and overall survival.

**Results:**

The study included 120 patients (40 in the CME group and 80 in the non-CME group). Lymph node yield was higher (29 *versus* 18, *P* = 0.006), the specimen length longer (322 *versus* 260 mm, *P* = 0.001) and median operative time was significantly longer (180 *versus* 130 min, *P* < 0.001) with robotic CME *versus* laparoscopy, respectively. Duration of hospital stay was longer with robotic CME, although not significantly (median 6 *versus* 5 days, *P* = 0.088). There were no significant differences in R0 resection rate, complications, readmission rates and local recurrence. A trend in survival benefit with robotic CME for disease-free (*P* = 0.0581) and overall survival (*P* = 0.0454) at 3 years was documented.

**Conclusion:**

Robotic CME with CVL is feasible and, although currently associated with a longer operation time, it provides good specimen quality, higher lymph node yield and acceptable morbidity, with a disease-free survival advantage.

## Introduction

Total mesorectal excision (TME) has resulted in improved local recurrence rates after Heald’s principles were accepted as the standard of care^1^. The concept of complete mesocolic excision (CME) of colonic cancers has gained variable acceptance across the surgical community. This is especially true for right colonic tumours, where the optimal extent and radicality of resection still remain unclear. Hohenberger applied the basic principles of total TME to the right colectomy, consisting of preserving the embryological mesocolic plane, dissecting the vessels centrally at the superior mesenteric artery (SMA) and vein, and extending the lymphadenectomy along the superior mesenteric vessels (SMV), including the infrapancreatic and gastroepiploic arcade nodes^2^. At least when compared to a historical cohort, lower recurrence rates and even improved survival were demonstrated^3^^,^^4^. Other authors independently analysed Erlangen specimens, confirming that CME surgery results in a higher mesocolic plane rate, with a greater area of mesentery containing a higher lymph node yield (LNY) and a larger overall specimen than standard UK and Danish cancer resection operations^5^.

However, the Hohenberger procedure includes Kocherization of the pancreatic head and lymphadenectomy around central vessels, which markedly increase its complexity. While the Hohenberger procedure was originally described using an open approach, modifications have enabled adoption of laparoscopy and decreased morbidity^6^^,^^7^. Commonly, the Kocherization and lymphadenectomy beyond the central middle colic vein are omitted. Still, the laparoscopic procedure remains technically challenging, which raises the question whether robotic surgery may facilitate adherence to the principles of CME, especially regarding central vascular ligation (CVL). In addition to conventional laparoscopy, the robotic system offers a surgeon-guided stable camera platform, providing an excellent three-dimensional view, seven degrees of freedom of the instrumentation, tremor filtering and individualized ergonomics. While robotic right colectomy with CME has recently been reported^8^, there are sparse data available on robotic CME when it comes to the technical aspects of central vascular dissection. This report aimed to assess the technical aspects and short-term and oncological outcomes of robotic right colectomy with CME and CVL for clinically node-positive right colonic cancers and cancers of the hepatic flexure/transverse colon.

## Methods

### Patients

Consecutive patients who underwent robotic right or extended right hemicolectomy with CME and CVL were included in this case series. The data were obtained from a prospectively collected and maintained, ethics committee-approved colorectal cancer database. Data were retrospectively analysed to assess the feasibility and safety of robotic right colectomy for tumours of the right colon. Patients were included if presenting with tumours of the hepatic flexure and transverse colon, where lymphatic drainage was along the right colic or middle colic vessels. Patients with cN1–2 disease on staging scans and aged 18–70 years were considered suitable for CME right or extended right hemicolectomy. Radiological criteria for nodal involvement included size greater than 10 mm, internal heterogeneity and an irregular border. Patient demographics, intraoperative details and postoperative outcomes were analysed.

In a subset analysis, the pilot group of CME cases was compared to a 2 : 1 propensity-matched laparoscopic right colectomy group, where no CVL or central lymphadenectomy was routinely performed. For propensity score-matching, all patients who underwent laparoscopic right-sided resections for colonic cancers at the authors’ institute between 2007 and 2017 were included. The comparative CME group was operated on between 2014 and 2017.

### Outcome measures

The primary outcomes for the study were total lymph node harvest and length of specimen. Secondary outcomes were operative time, postoperative complications (according to Clavien-Dindo classification), and disease-free (first recurrence after surgical resection (DFS)) and overall survival (any cause of death after surgical resection (OS)).

Pathological examinations of the resected specimens, including the number of harvested lymph nodes, were a particular focus as a surrogate for adequacy of oncological tumour clearance.

**Fig. 1:**
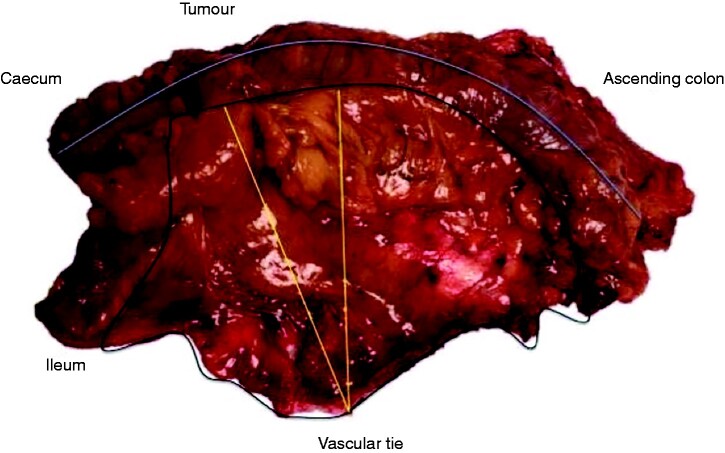
Specimen after complete mesocolic excision surgery for ascending colon cancer

Tissue measurements were taken by the pathologists on formalin-fixed specimens, and standard histopathology techniques without ancillary methods were used to assess the number and involvement of lymph nodes (*Fig. 1*). Central nodes were not assessed separately in this study. Patients were followed up with annual CT scan of chest, abdomen and pelvis for 5 years and a colonoscopy in the first 3 years after surgery. Recurrences were diagnosed based on radiology findings on the CT scan and with tissue biopsy where appropriate along with tumour markers (carcinoembryonic antigen). Local recurrences were defined as either luminal at the site of anastomosis, mesenteric or omental and peritoneal with carcinomatosis.

### Robotic surgical technique

Patients were placed in a modified Lloyd–Davis position and secured on an antislip mattress. Procedures were carried out using a da Vinci^®^ Si or X robotic surgical system (Intuitive Surgical, California, USA). Bony landmarks, namely the xiphisternum (XS), pubic symphysis (PS), costal margins on both sides and both anterior superior iliac spines (ASIS), were marked. A midline was drawn from XS to PS and the midpoint marked. Both ASIS were connected to the midpoint, forming the spinoumbilical line (SUL) on both sides. A line was drawn about 8 cm on either side of the midline, which constituted the midclavicular line (MCL).

**Fig. 2:**
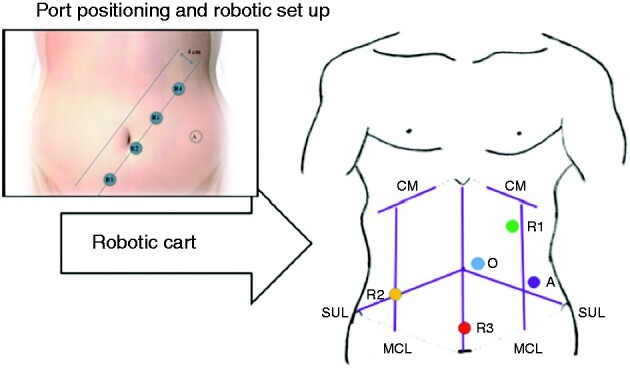
Robotic complete mesocolic excision (CME) surgery **a** Robotic set-up for CME/central vascular ligation right colectomy using Si System. **b** Port placement when using the DaVinci^®^ X system. R1, R2 and R3, robotic ports; A, assistant port; O, Optical port; CM, costal margin, MCL; midclavicular line; SUL, spinoumbilical line.

- Si System. The optical port was placed about 2 cm to the left of the midpoint on the midline. The first robotic port (R1) was marked in the left upper quadrant, 2 cm medial to the left MCL and 4–6 cm below the left costal margin. This point was also used for insertion of the Veress needle for creating the pneumoperitoneum. The second robotic port (R2) was marked at the point of transection of the right SUL and MCL. The third robotic port (R3) was marked in the suprapubic area, 8–10 cm below the optical port. The assistant port was nearly a mirror image of the R2 on the right side (*Fig. 2*). The minimum distance between the ports and the distance from the camera port should be 8 cm (*Fig. 2a*).
- X/Xi System. The intersection of the midclavicular line was joined to the midpoint of the inguinal ligament; on this line, four robotic ports were placed at least 6 cm apart and at least 3 cm away from the bony landmarks (*Fig. 2b*). An assistant port was placed in the left iliac fossa. The camera was placed in R1 or R2, depending upon the stage of the procedure. The procedure started with the creation of the pneumoperitoneum with a Veress needle at the marked site for R4. After inserting all ports, the patient was placed in a 15° Trendelenburg position and a 15° left tilt.

The first part of the procedure was the laparoscopic set-up, which includes exposure of the transverse colon by pushing the omentum over the transverse colon to the upper abdomen. The small bowel was brought to the left side, exposing the root of the mesenteric vessels. If necessary, a swab was used to keep the small bowel loops out of the operating field. The robotic cart was docked from the right side of the patient midway between the ASIS and CM.

Dissection started at the root of the SMV pedicle, where the peritoneal layer covering the mesentery was opened using monopolar diathermy. The mesenteric fat was dissected to expose the SMV alongside the SMA. In patients with high BMI the use of robotic ultrasound probe was standard to identify the exact location of SMV, as previously reported and demonstrated *Video S1*. Ileocolic and right colic vessels were ligated at their origin using robotic hemolock clips. The dissection was continued cranially and led to the origin of the middle colic vessels. The middle colic vein was divided at its origin. The middle colic artery at its origin, or only the right branch, was divided depending upon the location of the tumour. The Henle’s trunk was dissected and the colic branch ligated. The right gastroepiploic vein was dissected of lymphatic tissue or ligated and resected centrally. Anatomical variations were common at this point, which had to be considered. Medial to lateral dissection was carried out between the visceral and parietal peritoneum, exposing the head of pancreas completely. After performing the subileal, lateral and hepatic flexure mobilization, the small bowel mesentery and corresponding part of the omentum were transected using a robotic vessel sealer. The gastrocolic omentum was divided using a vessel sealer, sparing the gastroepiploic arcade. Kocherization of the duodenum was not performed routinely unless lymphadenopathy was seen in the retropancreatic nodal station on the CT scan. A bolus of 3 ml indocyanine green was administered intravenously to assess the vascularity of the transresection sites of the transverse colon and terminal ileum. Three firings of a liner 60 mm stapler were used for transection of the transverse colon, terminal ileum and intracorporeal ileocolic anastomosis. The enterotomy was closed with a 3/0 vicryl running suture in two layers, and the specimen extracted through a Pfannenstiel incision.

### Laparoscopic surgical technique

Surgeons had had an established practice for over 5 years and were trainers in the LapCo training programme. A standard four-port laparoscopic right colectomy was carried out. Ileocolic, right colic and middle colic vessels were ligated at origin between the clips. Routine exposure of SMV was not carried out. D2 lymph node dissection was carried out with ligation of vessels between hemolock clips and medial to lateral mobilization of the right colon. A midline extraction was made and an extracorporeal stapled side-to-side anastomosis was fashioned.

### Statistical analysis

To decrease variation in baseline characteristics between robotic and laparoscopic cases, nearest neighbour 2 : 1 propensity score-matching without a caliper was applied to minimize selection bias. In the common sample, co-variables in regression analysis with method as the binary outcome variable to obtain the propensity scores were age, sex, ASA grade, BMI and preoperative TNM stage. Cases from the conventional laparoscopic pool with the nearest propensity scores to the robotic cases were 2 : 1 matched. A mean standardized difference of the co-variables between the matched groups of less than 0.25 (IdI = 0.25) was considered a balanced match. Conditional independence of the outcome from treatment status after controlling for the co-variables used for matching was assumed. Furthermore, for each set of values of those co-variables, a positive probability of being both treated and not treated was assumed (common support) and tested graphically. Data were expressed as median with interquartile ranges. Intergroup comparisons were made using a Mann–Whitney U test for continuous variables or χ^2^ or Fishers exact test for categorical variables. *P* < 0.05 was considered significant. A time-to-event analysis was performed using the Kaplan–Meier method. Comparison of the survival and local recurrence rates between the two surgical methods was analysed by the log-rank test. All statistical analyses, including propensity score matching, were performed using SPSS^®^ Statistics for Windows, Version 23 (IBM, New York, USA).

## Results

### Patients

The study population consisted of 40 robotically operated and 282 laparoscopically operated right colectomy cases. Following propensity score matching 120 patients were included. A total of 40 patients underwent robotic CME (D3 lymphadenectomy) for right**-**sided tumours over a 3-year period (21 females; median age 68 (range 34–80) years; median BMI 26 kg/m^2^) (*Table 1*). Tumours were distributed in the caecum (4 patients), ascending colon (18 patients), hepatic flexure (12 patients) and transverse colon (6 patients). Eighty per cent of patients underwent right hemicolectomy and the remaining 20 per cent had extended right hemicolectomy. The non-CME, propensity score-matched group enlisted 80 patients (43 females; median age 71 (37–82) years; median BMI 28 kg/m^2^) treated using laparoscopy.

**Table 1:** Patient characteristics

Characteristic	CME, robotic (*n* = 40)*	Non-CME, laparoscopic (*n* = 80)*	*P*	SMD	VR
**Age^†^**	69 (34–80)	71 (37–82)	0.976^‡^	−0.700	1.17
**Gender**			0.897^§^		0.99
Male	19 (34)	37 66)		−0.056	
Female	21 (33)	43 (67)		0.056	
**ASA**			0.094^§^		0.93
1	5 (63)	3 (38)		0.135	
2	28 (35)	53 (65)		0.132	
3	7 (23)	24 (77)		−0.197	
**BMI^†^**	26 (20–37)	28 (19–47)	0.568	−0.143	0.84
**Preoperative T stage**			0.098^‡^		4.12
T1/ T2	9 (22)	32(78)		0.554	
T3/T4	28 (37)	48 (63)		0.528	

* Values in parentheses are percentages unless indicated otherwise;

† values are median (i.q.r.). SMD: standardized mean difference; VR: variance ratio, VR expected range is (0.52;1.92); CME, complete mesocolic excision.

‡ Mann-Whitney U test, §χ^2^ test.

### Lymph node harvest

There was a significantly greater increase in LNY in the CME group (29 *versus* 18; *P* = 0.006) (*Table 2*). R0 resection rates and local recurrence were similar across both groups; however, there was a higher distant metastasis rate in the non-CME group (19/80 *versus* 3/40; *P* ≤ 0.026). The majority of tumours (CME, 77 per cent; non-CME, 60 per cent) were T3/T4. The use of adjuvant chemotherapy was similar between groups (CME, 31 per cent patients; non-CME, 30 per cent).

**Table 2:** Oncological data

	CME, robotic (*n* = 40)*	Non-CME, laparoscopic (*n* = 80)*	*P*
**pT stage**			0.098^‡^
T1/T2	9 (23)	32 (40)	
T3/T4	31 (78)	48 (60)	
N0	25 (63)	46 (58)	
N1	5 (13)	22 (28)	
N2	10 (25)	12 (15)	
**Lymph node harvest^†^**	29 (19–60)	18 (8–53)	0.006^§#^
**R0 resection**	39 (98)	80 (100)	0.327^¶^
**Adjuvant chemotherapy**	13 (33)	24 (30)	0.847^¶^
**Local recurrence**	0	5 (6)	0.102^‡^
**Distant recurrence**	3 (8)	19 (24)	0.026^¶#^

* Values in parentheses are percentages unless indicated otherwise; ^†^ values are median (i.q.r.).

‡ Fisher’s exact test,

§ Mann–Whitney U test,

¶ χ^2^ test.

# Statistically significant at 5%. CME, complete mesocolic excision.

The median length of the specimen was significantly longer in the CME group (322 *versus* 260 mm; *P* = 0.001). The median small bowel and large bowel length and distance of tumour from distal resection margin was 100 (i.q.r. 35–170) mm, 210 (i.q.r. 105–315) mm and 55 (i.q.r. 35–75) mm, respectively, for the CME group *versus* 70 (i.q.r. 30–110) mm, 180 (i.q.r. 95–265) mm and 50 (i.q.r. 30–60) mm for the non-CME group (*Table 3*).

**Table 3:** Specimen size

Characteristics	CME, robotic (*n* = 40)*	Non-CME, laparoscopic (*n* = 80)*	*P*
**Length of specimen (mm)**	322 (300–390)	260 (202–320)	<0.001^†‡^
**Length of small bowel removed (mm)**	100 (35–170)	70 (30–110)	<0.001^†‡^
**Length of colon removed (mm)**	210 (105–315)	180 (95–265)	<0.001^†‡^
**Distance of tumour from distal margin**	55 (35–75)	50 (30–60)	0.821

* Values are median (i.q.r.).

† Mann–Whitney U test.

‡ Statistically significant at 5%. CME, complete mesocolic excision.

### Intraoperative outcomes

Operative times were longer in the CME group compared to the non-CME group (180 min *versus* 130 min, *P* ≤ 0.001) (*Table 4*). Similarly, the median duration of stay was longer in the CME group, but did not reach statistical significance (6 *versus* 5 days, *P* = 0.088). Median blood loss was lower in the CME group (10 ml) than the non-CME group (50 ml; *P* = 0.569). Four patients in the non-CME group were converted to open surgery due to technical and oncological reasons; there were no conversions in the CME group. Plane of surgical excision was not available for the laparoscopic cohort; with the implementation of CME programme, however, the pathologists routinely reported the grade of mesocolic excision. Over 92 per cent of patients in the CME group had a mesocolic plane of excision.

**Table 4:** Intraoperative and postoperative data

	CME, robotic (*n* = 40)*	Non-CME, laparoscopic (*n* = 80)	P value
**Docking time (min)**	8 (5–25)	n.a.	
**Total operating time (min)**	180 (128–300)	130 (90–280)	<0.001^¶^
**Console time (min)**	135 (105–270)	n.a.	
**Blood loss (mm)**	10 (0–20)	50 (10–250)	0.569
**Conversion^†^**	0	4 (5)	0.186^‡^
**Length of stay (days)**	6 (3–14)	5 (2–41)	0.088^§^
**Complications^†^**			
Grade III/IV	2 (5)	4 (5)	0.337^‡^
Grade I/II	4 (10)	10 (13)	0.229^‡^
**Readmission in 30 days^†^**	3 (8)	6 (7)	0.545^‡^

* Values are median (i.q.r.) except where indicated; ^†^ values in parentheses are percentages.

‡ Fisher’s exact test,

§ Mann–Whitney U test.

¶ Statistically significant at 5%. CME, complete mesocolic excision; n.a., not applicable.

### Complications and postoperative outcomes

There was no difference in minor (Clavien-Dindo grade I/II) and major (Clavien-Dindo grade III/IV) complications or readmission rate between groups (*Table 4*). In the CME group, one patient had a radiological leak that did not require reintervention. Minor complications included wound infection (1 patient), ileus (2 patients) and rectal bleed (3 patients). There was no 30- or 90-day mortality.

Median duration of hospital stay was longer with robotic CME group, although this was not significant (median 6 *versus* 5 days, *P* = 0.088).

### Oncological outcomes

**Fig. 3:**
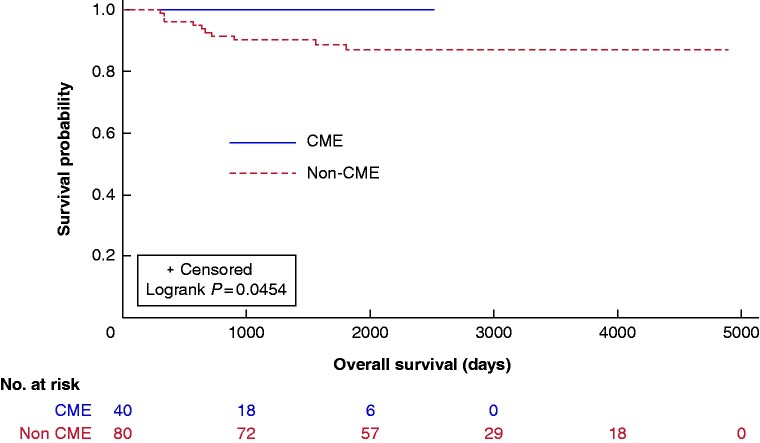
Kaplan–Meier plots of overall survival in complete mesocolic excision (CME) and non-CME cases

**Fig. 4:**
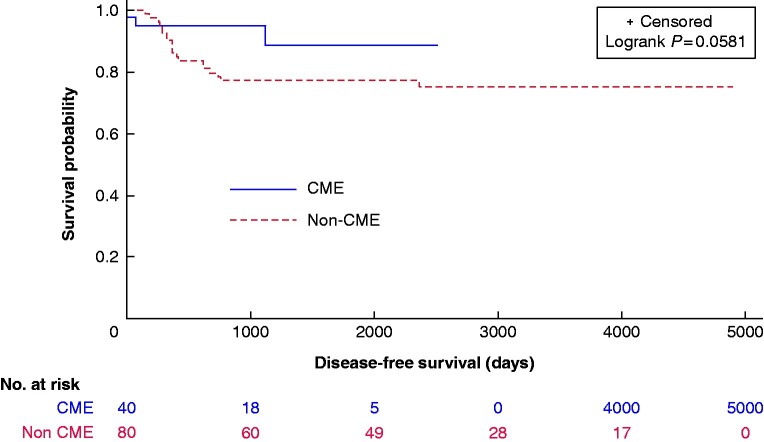
Kaplan–Meier plots of disease-free survival in complete mesocolic excision (CME) and non-CME cases

There was a trend towards improved DFS and OS in the CME group (*Figs. 3, 4*). The median 3-year DFS and OS were 90 and 99 per cent for CME and 78 and 90 per cent for the non-CME group.

## Discussion

About 15 per cent of cancers confined to the colonic wall will have metastatic spread to lymph nodes^9^. A suboptimal lymphadenectomy, spillage of malignant cells or an R1 resection would undoubtedly increase the risk of local recurrence. The average risk of recurrence after colon cancer surgery is around 11 per cent at 5 years after potentially curative surgery, and is considered highest for hepatic flexure tumours^10^. The number of lymph nodes harvested and mesenteric/mesocolic specimen size increase with CME, as shown in the Erlangen series. In the present study, an increased lymph node harvest was demonstrated in the robotic CME group compared to the standard laparoscopic right colectomy group, along with an increase in the specimen size and bulk. The nodal harvest in colorectal surgery is important for staging and therefore decision making for adjuvant therapy^11^. Although fewer than 12 lymph nodes have been shown to be associated with poorer prognosis, no further value has been reported for a higher number of lymph nodes^4^. More recently, a higher number of lymph nodes (more than 28) harvested in CME were reported to be associated with a better prognosis^12^. This may be because a higher LNY could lead to stage migration and consecutively to more intensive systemic therapy, associated with a better prognosis.

Shortcomings of conventional laparoscopic surgery found to be of particular relevance in the treatment of hepatic flexure and transverse colon cancers were a shorter vascular pedicle length and lower number of lymph nodes compared to open surgery, questioning the oncological adequacy^5^^,^^13^. Robotic-assisted CME, with its better technical capability of radical central lymph node and vascular dissection, is beneficial in potentially overcoming these limitations and leading to improved outcomes^14^. Although CME/CVL right hemicolectomy can also be achieved by conventional laparoscopy, a high level of laparoscopic expertise is mandatory to avoid compromise in difficult situations.

Outcomes following colonic cancer resection have been outpaced by the advancements in multidisciplinary rectal cancer treatment. International standardization of the surgical technique^15^, guided by high-resolution MRI scanning^16^ and supported by histopathological quality control, has enabled TME to greatly reduce local recurrence rates and improve overall survival^17^. The embryological anatomical planes that underpin TME principles extend proximally to include the entire colon^3^^,^^18^. Therefore, CME surgery should theoretically benefit patients with colonic cancer. The 3 year DFS and OS in this study was significantly better compared with the non-CME group and compares favourably with the published literature^2^^,^^19^.

CME surgery comprises two key components: preservation of the integrity of the visceral mesocolic fascia to reduce the chance of tumour cell dissemination, and CVL. Division of the supplying artery at its origin ensures that central lymph node groups receiving lymph from potentially involved pericolic nodes are excised, which may make the difference between a curative outcome and locoregional recurrence^8^. In contrast to mesorectal excision, there is still a lack of standardization of the CME technique, and the use of different terminology such as D3 lymphadenectomy, extended lymphadenectomy or radical right colectomy can be confusing^19^. Radical CME/CVL right colectomy has not gained widespread popularity due to the lack of evidence showing improved survival, as well as the potential morbidity resulting in longer hospital stay^20^. The latter is confirmed in this series by the longer median length of stay by 1 day in the CME group. In this series, however, there were no cases with major vascular injury or chylous ascites. In most cases, those complications can be avoided by choosing a dissection plane in front of the SMV, while leaving the neurolymphovascular tissue around the SMA in place. Starting the vascular dissection distally on the SMV is advisable, as even in case of damage at that level the vein can be safely ligated when local bleeding control is not otherwise possible. To facilitate localization of the distal SMV, especially in obese patients, the use of intraoperative ultrasound, which may further reduce the risk of inadvertent vascular injury, was recently reported^21^.

Laparoscopy is well established for standard right colectomy, while there is uncertainty regarding its use in CME/CVL surgery. The robotic approach for CME surgery for colon cancer is attractive because of its ergonomic advantages^18^; however, there are limited data published in this context^6^. CME with CVL exclusively for tumours of the hepatic flexure/transverse colon and node-positive right colonic tumours has not been reported. Purely laparoscopic approaches to radical right colectomy with CME have been described in the literature^22^^,^^23^. Robotic-assisted surgery has an advantage of accessing the target organ more precisely and from angles practically unattainable with laparoscopy. As a personal choice, an SMV first medial-to-lateral approach was preferred for dissection during a robotic CME, as it minimizes the risk of complications and bleeding by early exposure of the retroperitoneal structures and ligation of feeding vessels close to their origin; furthermore, there is less tumour manipulation^24^.

Limitations of this study include its retrospective nature, as selection bias may be a considerable confounder, although it has been minimized by propensity score matching. Furthermore, the laparoscopically experienced surgeons participating in this series may still be in their learning curve of robotic CME, and outcomes may improve further over time with the robotic method. Finally, two different operations (conventional *versus* CME/CVL right colectomy) and two different methods (laparoscopic *versus* robotic) were compared simultaneously, which compromises the evidence.

To date, no RCT has been published showing superiority of CME/CVL in right colonic cancers. Available cohort studies and meta-analyses suggest improved survival and lower recurrence rates with CME surgery^25^^,^^26^. However, further evidence is required to justify implementation of the technique in routine clinical practice^25^^,^^27^. Larger-scale studies and registry data are necessary before a strong recommendation for a wider adoption of the technique can be made.

## Supplementary Material

zrab016_Supplementary_Data
